# Kernel-Based Relevance Analysis with Enhanced Interpretability for Detection of Brain Activity Patterns

**DOI:** 10.3389/fnins.2017.00550

**Published:** 2017-10-06

**Authors:** Andres M. Alvarez-Meza, Alvaro Orozco-Gutierrez, German Castellanos-Dominguez

**Affiliations:** ^1^Automatics Research G., Universidad Tecnologica de Pereira, Pereira, Colombia; ^2^Signal Processing and Recognition G., Universidad Nacional de Colombia, Manizales, Colombia

**Keywords:** relevance analysis, kernel method, brain activity, motor imagery, epileptic seizure detection

## Abstract

We introduce *Enhanced Kernel-based Relevance Analysis* (EKRA) that aims to support the automatic identification of brain activity patterns using electroencephalographic recordings. EKRA is a data-driven strategy that incorporates two kernel functions to take advantage of the available joint information, associating neural responses to a given stimulus condition. Regarding this, a *Centered Kernel Alignment functional* is adjusted to learning the linear projection that best discriminates the input feature set, optimizing the required free parameters automatically. Our approach is carried out in two scenarios: (i) feature selection by computing a relevance vector from extracted neural features to facilitating the physiological interpretation of a given brain activity task, and (ii) enhanced feature selection to perform an additional transformation of relevant features aiming to improve the overall identification accuracy. Accordingly, we provide an alternative feature relevance analysis strategy that allows improving the system performance while favoring the data interpretability. For the validation purpose, EKRA is tested in two well-known tasks of brain activity: motor imagery discrimination and epileptic seizure detection. The obtained results show that the EKRA approach estimates a relevant representation space extracted from the provided supervised information, emphasizing the salient input features. As a result, our proposal outperforms the state-of-the-art methods regarding brain activity discrimination accuracy with the benefit of enhanced physiological interpretation about the task at hand.

## 1. Introduction

The electroencephalogram (EEG) is the electrical activity of neurons in subcortical structures recorded by a noninvasive electrode array placed on the brain scalp surface. Because of their high temporal resolution and low cost, the biological EEG recordings have been found to be effective in many neurophysiological applications related to brain-computer interfaces (Nicolas-Alonso and Gomez-Gil, 2012), automated diagnosis of neurological diseases like epilepsy (Faust et al., 2015), neuromarketing (Vecchiato et al., 2015), and sensorimotor restoration (Pisotta et al., 2015), just to mention a few examples. As part of the data analysis in these applications, however, it is essential to manage massive amounts of the input feature space that frequently holds large dimensions (~10^2^–10^3^ features) and limited numbers of samples (up to a few dozen) (Wang et al., 2015). As a consequence of high-dimensional data, most of the machine learning algorithms may cause inefficiency and low accuracy (Fang et al., 2016).

In practice, feature extraction from EEG recordings is a particular way of data reduction, which is carried out to represent the brain states, enabling an efficient pattern identification and translation of mental states. For the goal to feed the machine learning classifiers, a variety of EEG features may be extracted. Thus, the linear extraction methods are widespread, which are more applicable to stationary signal processing, such as linear Fourier-based spectral analysis, auto-regressive models, Time-Frequency Distributions (Al-Fahoum and Al-Fraihat, 2014). In the case of sudden and transient signal changes, more popular methods are Wavelet decomposition (Duque-Muñoz et al., 2014) and empirical mode decomposition (Zhang et al., 2016). Due to the high non-stationary and non-linearity of EEG data, nonlinear extraction methods (e.g., Entropic and complexity measures) are employed that usually provide a higher classification accuracy, but at the cost of increased computational burden (Acharya et al., 2012; Chen et al., 2015), without mentioning their complicated suitability in multi-channel setups (Chen et al., 2016). Therefore, each method has particular strengths and weaknesses, meaning that the effectiveness of each feature extraction method depends on the application.

### 1.1. Related work

There are two alternative approaches to addressing the problem of a large amount of EEG data (Naeem et al., 2009): (i) *Channel selection* that intends to choose a subset of electrodes contributing the most to the desired performance. Besides of avoiding redundancy of non-focal/unnecessary channels, this procedure makes visual EEG monitoring more practical when the number of needed channels becomes very few (Alotaiby et al., 2015). A significant disadvantage of decreasing the number of EEG channels is the unrealistic assumption that cortical activity is produced by EEG signals coming only from its immediate vicinity (Haufe et al., 2014). (ii) *Dimensionality Reduction* that projects the original feature space into a smaller space representation, aiming to reduce the overwhelming number of extracted features (Birjandtalab et al., 2017).

Although either approach to dimensionality reduction can be performed separately, there is a growing interest in minimizing together the number of channels and features to be handled by the classification algorithms (Martinez-Leon et al., 2015). According to the way the input data points are mapped into a lower-dimensional space, dimensionality reduction methods can be categorized as linear or non-linear. The former approaches (like Principal Component Analysis (Zajacova et al., 2015), Discriminant and Common Spatial Patterns (Liao et al., 2007; Zhang et al., 2015), and Spatio-Spectral Decomposition) are popular choices for either EEG representation case (channels or features) with the benefit of computational efficiency, numerical stabilization, and denoising capability. Nevertheless, they face a deficiency, namely, the feature spaces extracted from EEG signals can induce significant and complex variations regarding the nonlinearity and sparsity of the manifolds that hardly can be encoded by linear decompositions (Sturm et al., 2016). Moreover, based on their contribution to a linear regression model, linear dimensionality reduction methods usually select the most compact and relevant set of features, which might not be the best option for a non-linear classifier (Adeli et al., 2017).

In turn, the non-linear mapping can more precisely preserve the information about the local neighborhoods of data-points by introducing either locally linearized structures or pairwise distances along the subtle non-linear manifold, attempting to unfold more complex high-dimensional data as separable groups (Lee and Verleysen, 2007). Among machine learning approaches to dimensionality reduction, the Kernel-based analysis is promising because of the following properties (Chu et al., 2011): (i) kernel methods apply a non-linear mapping to a higher dimensional space where the original non-linear data become linear or near-linear. (ii) The Kernel trick decreases the computational complexity of high dimensional data as the parameter evaluation domain is lessened from the explicit feature space into the Kernel space. In practice, an open issue is the definition of the kernel transformation that can be more connected with the appropriate type of application nonlinearity (Zimmer et al., 2015). Nevertheless, more efforts are spent in the development of a metric learning that allows a kernel to adjust the importance of individual features of tasks under consideration, usually exploiting a given amount of supervisory information (Hurtado-Rincón et al., 2016). Hence, the kernel-based relevance analysis can handle the estimated weights to highlight the features or dimensions relevant for improving the classification performance (Brockmeier et al., 2014).

### 1.2. Our contribution

Devoted to channel selection and dimension reduction of EEG data, some issues remain open in employing Kernel-based metric learning algorithms: (i) Adaptation to the complex neural relationships is far from being an easy task, in particular, in taking advantage of the supervised information by non-linear methods (Fukumizu et al., 2004). (ii) A direct physiological interpretability from a non-linear-based mapping is not always possible. (iii) The selected or reduced feature sets with the smallest size can provide a high rate of false alarms and missed detections. This situation hinders a solid interpretation of the mechanisms underlying the problem (Gajic et al., 2014). (iv) The computational complexity is a strong constraint because of the extensive processing time and parameter tuning (mainly performed using heuristic methods). Thus, there is a need for identifying the most discriminating features by finding a trade-off between system complexity and accuracy (Bhattacharyya et al., 2014). (v) High variability of channel performance across the subjects (Feess et al., 2013). Due to the personal peculiarities, the high inter-subject variability poses one of the biggest challenges in brain activity identification.

In this work, we develop a kernel-based approach for enhanced feature relevance analysis, termed *Enhanced Kernel Relevance Analysis* (EKRA), aiming to identify brain activity patterns automatically. In particular, the proposed relevance analysis comprises two kernel functions to take advantage of the actual joint information, attaching neural responses to a given stimulus/conditions. In this regard, we employ a *Center Kernel Alignment* (CKA)-based functional to learn a linear projection that encodes all discriminative input features, benefiting from the non-linear notion of similarity behind the studied kernels (Cortes et al., 2012). The present approach is an extension of our previous *Kernel Relevance Analysis* strategy describe in (Arias-Mora et al., 2015; Hurtado-Rincón et al., 2016). In particular, EKRA can be implemented as both feature selection (EKRA-S) and enhanced feature selection (EKRA-ES) tool. The former introduces a feature relevance vector index to quantify the contribution of each input feature for discriminating the possible stimulus/conditions. The latter adapts the relevance index vector to compute a representation space favoring the brain activity patterns separability without redundancy. Besides, an iterative gradient descent optimization is applied to calculate the EKRA projection matrix and the required kernel free parameters. From the attained results we conclude that EKRA allows finding a suitable feature representation space by ensuring the identification of brain activity patterns, at the same time, favoring the physiological interpretation of the studied phenomenon. Indeed, our proposal outperforms state-of-the-art approaches that carry out the multivariate feature selection and dimensionality reduction.

The rest of the paper is organized as follows: In Section 2, we develop the theoretical background of the introduced EKRA. Section 3 describes the experimental set-up for Identification of Brain Activity Patterns, Section 4 discussed the obtained results, and the concluding remarks are outlined finally in Section 5.

## 2. Methods

### 2.1. Feature extraction using centered kernel alignment

Given that neural responses to brain activity tasks are contained in a domain X, a kernel $\kappa_{X}:X\times X\to \mathbb{R}$ is assumed to be a positive-definite function, which reflects an implicit mapping $\phi :X\to H_{X}$, associating an element x∈X with the element $\phi \left(x\right)\in H_{X}$ that belongs to the Reproducing Kernel Hilbert Space (RKHS), $H_{X}$. For associating elements, kernel functions are built using several bivariate measures of similarity, which are based on the inner product between samples contained in a RKHS. Although, various functions have been tested, the Gaussian function is preferred in pattern classification and machine learning applications since it aims at finding an RKHS with universal approximating ability, not to mention its mathematical tractability (Liu et al., 2011; Brockmeier et al., 2013). For Gaussian kernels, each pairwise similarity distance between samples $x,x^{\prime }\in X$ is computed as follows (Wang et al., 2016):

(1) $$\kappa_{X}(x,x\prime ;\sigma )=\exp(-d^{2}(x,x\prime )/2\sigma^{2})$$

where d(·,·):X×X↦ℝ is a distance operator defined on the neural response domain X, and σ ∈ ℝ^+^ is the kernel bandwidth that rules the observation window within the similarity distance is assessed. Likewise, on the neural stimulus/condition space L, which contains the target membership of the neural responses (e.g., brain activity task labels), we also set a positive definite kernel $\kappa_{L}:L\times L\mapsto H_{L}$ that performs the a non-linear mapping $\varphi :L\mapsto H_{L}$. Thus, provided a sample set $l,l^{\prime }\in L$, the pairwise similarity for neural stimuli/conditions is defined like $\kappa_{L}\left(l,l^{\prime }\right)=\pi_{ll^{\prime }}$, where delta function is $\pi_{ll^{\prime }}=1$ if *l*=*l*′, otherwise, $\pi_{ll^{\prime }}=0$.

It is worth noting that each defined above kernel reflects a different notion of similarity. So, we apply two kernel functions sequentially to assess the shared information between the neural responses to a particular stimulus/condition and the corresponding labels. Therefore, we must still evaluate how well the kernel function, κ_*X*_, matches the target kernel of labels, κ_*L*_. To this end, we introduce a kernel target alignment to appraise the similarity between a couple of characterizing kernel functions, employing the inner product of both kernels to estimate the dependence between the jointly sampled data (Gretton et al., 2005). Thus, the statistical alignment between κ_*X*_ and κ_*L*_ (termed *Centered Kernel Alignment* – CKA) is computed as their normalized inner product averaged across all realization pairs as below (Cortes et al., 2012):

(2) $$\rho (\kappa_{X},\kappa_{L})=\frac{{𝔼}_{xx\prime ,l,l\prime }\{{\bar{\kappa }}_{X}(x,x\prime ;\sigma ){\bar{\kappa }}_{L}(l,l\prime )\}}{\sqrt{{𝔼}_{xx\prime }\{{\bar{\kappa }}_{X}^{2}(x,x\prime ;\sigma )\}{𝔼}_{ll^{\prime }}\{{\bar{\kappa }}_{L}^{2}(l,l\prime )\}}},$$

where notation 𝔼_*x*_{·} stands for the expectation value operator calculated over the random variable x∈X, and notation $\bar{\kappa }\left(\cdot \right)$ stands for the centered version of each kernel that is estimated as follows, respectively:

(3a) $$\begin{array}{l} {\bar{\kappa }}_{X}(x,x\prime ;\sigma )=\kappa_{X}(x,x\prime ;\sigma )-{𝔼}_{x\prime }\{\kappa_{X}(x,x\prime ;\sigma )\}\\ \;-{𝔼}_{x}\{\kappa_{X}(x,x\prime ;\sigma )\}+\;{𝔼}_{xx\prime }\{\kappa_{X}(x,x\prime ;\sigma )\}, \end{array}$$

(3b) $$\begin{array}{l} {\bar{\kappa }}_{L}(l,l\prime )=\kappa_{L}(l,l\prime )-{𝔼}_{l\prime }\{\kappa_{L}(ll\prime )\}-{𝔼}_{l}\{\kappa_{L}(l,l\prime )\}\\ \;+{𝔼}_{ll\prime }\{\kappa_{L}(l,l\prime )\}. \end{array}$$

Hence, the larger the similar pairs between the X and L spaces, the higher their CKA alignment value ρ∈ℝ[0, 1].

In practice, provided an input representation set ***X***∈ℝ^*N*×*P*^ (with $X\subset X\in {\mathbb{R}}^{P}$) together with a vector of respective stimulus/condition labels ***l***∈ℤ^*N*^ (l⊂L∈ℤ), we extract each characterizing kernel matrix, $K_{X}\in {\mathbb{R}}^{N\times N}$ and $K_{l}\in {\mathbb{R}}^{N\times N}$. The former matrix holds elements $k_{nn^{\prime }}^{X}=\kappa_{X}\left(x_{n},x_{n^{\prime }}\right)$ with $x_{n},x_{n^{\prime }}\in X$, while the latter matrix has elements $k_{nn^{\prime }}^{l}=\kappa_{L}\left(l_{n},l_{n}^{\prime }\right)$ with $l_{n},l_{n^{\prime }}\in l$ (*n, n*′∈ℕ[1, *N*]), where *N*∈ℕ is the number of neural response samples and *P*∈ℕ is the amount of estimated features. Using Equation (3), then, the empirical estimate for the CKA alignment can be calculated as follows:

(4) $$\hat{\rho }(K_{X},K_{l})=\frac{{\langle {\bar{K}}_{X},{\bar{K}}_{l}\rangle }_{\text{F}}}{\sqrt{{\langle {\bar{K}}_{X},{\bar{K}}_{X}\rangle }_{\text{F}}{\langle {\bar{K}}_{l},{\bar{K}}_{l}\rangle }_{\text{F}}}},$$

where notation $\bar{K}$ stands for the centered kernel matrix calculated as $\bar{K}=\tilde{I}K\tilde{I}$, where $\tilde{I}=I-1^{⊤}1/N$ is the empirical centering matrix, ***I***∈ℝ^*N*×*N*^ is the identity matrix, and **1**∈ℝ^*N*^ is the all-ones vector. Notation 〈·, ·〉_F_ denotes the matrix-based Frobenius norm.

Consequently, the alignment in Equation (4) is a data-driven estimator that, from the input matrix ***X***, allows quantifying the similarity between the input sample space and the stimulus/condition labels ***l***.

### 2.2. Enhanced kernel-based relevance analysis

For the implementation of selected Gaussian kernel κ_*X*_, we further rely on the Mahalanobis distance to perform the pairwise comparison between samples ***x***_*n*_ and $x_{n^{\prime }}$. Namely, the distance function in Equation (4)is fixed as follows:

(5) $${\text{d}}_{A}^{2}(x_{n},x_{n^{\prime }})=(x_{n}-x_{n^{\prime }})AA^{⊤}{\left(x_{n}-x_{n^{\prime }}\right)}^{⊤},\forall n,n^{\prime }\in N$$

where matrix ***A***∈ℝ^*P*×*M*^ holds the linear projection in the form ***y***_*n*_ = ***x***_*n*_***A***, with $y_{n}\in {\mathbb{R}}^{M},$ being ***AA***^⊤^ the corresponding inverse covariance matrix, assuming *M*≤*P*. Therefore, intending to compute the projection matrix ***A***, the formulation of CKA-based optimizing function in Equation (4) can be integrated into the following kernel-based learning algorithm:

(6) $$\hat{A}=\arg\;\underset{A}{\max}\log(\hat{\rho }(K_{X}(A,\sigma ),K_{l})),$$

where the logarithm function is here used just for mathematical convenience. Note that we highlight the dependence of the kernel matrix ***K***_***X***_(***A***, σ) concerning both the projection matrix ***A*** and the Gaussian kernel bandwidth σ. In this work, we propose to solve the optimization problem in Equation (6) using a recursive solution based on the well-known gradient descent approach (See Appendix A in Supplementary Material for further details).

After estimation of the projection matrix $\hat{A}$, we assess the relevance of *P* input features extracted from ***X***. To this end, we assume that the most contributing features should have higher values of similarity relationship with the provided neural stimuli/condition. Specifically, the CKA-based relevance analysis calculates the feature relevance vector index, ϱ∈ℝ^*P*^, having elements $\varrho_{p}\in {\mathbb{R}}^{+}$ that allow measuring the contribution of each *p*-th input feature in building the projection matrix $\hat{A}$. So, we use the stochastic measure of variability proposed in (Daza-Santacoloma et al., 2009) as follows:

(7) $$\varrho_{p}={𝔼}_{m\in M}\{|a_{pm}|\},$$

where $a_{pm}\in \hat{A}$ indexes every element of matrix $\hat{A}$ (*p*∈*P*, *m*∈*M*).

Therefore, this improvement of the feature extraction using centered kernel alignment (termed *Enhanced Kernel-based Relevance Analysis*– EKRA) counts on the interpretability provided by its two central stages: (i) Seeking a feature relevance vector ϱ, relying on the averaged weight magnitudes of the CKA-based rotation matrix that is directly related to the separability contribution of *p*-th input feature (see Equation 7). In fact, the larger the ϱ_*p*_ value, the higher the participation of *p*-th feature to match the neural responses in the input space with the stimulus/condition label set. So, we compute the matrix $X^{\prime }\in {\mathbb{R}}^{N\times M_{S}}$ to select *M*_*S*_<*P* features, applying the threshold value of separability contribution: ϱ_*p*_>𝔼_*m*∈*M*_{ϱ_*p*_}. As a result, EKRA allows explaining the measured discriminating capability provided by each feature since the obtained relevance vector preserves the one-to-one relationship to the input space. (ii) Linear projection of the achieved CKA relevance subset that intends to enhance the stimulus/condition separability further, based on the explained discrimination (ruled by the introduced constraint ϱ_*p*_>𝔼_*m*∈*M*_{ϱ_*p*_}) that is measured by the input neural responses. Concerning this, the mapped feature matrix $Y\in {\mathbb{R}}^{N\times M_{E}}$ is calculated as: ***Y*** = ***X***′***A***′, where *M*_*E*_ is the resulted amount of relevant features, and $A^{\prime }\in {\mathbb{R}}^{M_{S}\times M_{E}}$ is a rotation matrix computed from ***X***′, using Equation (6) and under the assumption that *M*_*S*_<*M*_*E*_.

## 3. Experimental set-up

For identification of the tested brain activity patterns, we validate the proposed *Enhanced kernel-based relevance analysis* that appraises the following training stages: (i) Feature extraction from the preprocessed EEG recordings, (ii) EKRA computation for the extracted feature set, and (iii) Detection performance of brain patterns under stimuli/conditions.

### 3.1. Testing datasets and preprocessing

Intending to test two different tasks of brain activity, the validating experiments are carried out on each one of the following EEG databases:

***Motor Imagery Database* (MIDB)**^1^. This collection that is widely experimented in motor imagery tasks holds seven subjects with EEG signals recorded from 59 channels. Firstly, all recordings are submitted to a bandpass filter with bandwidth ranging from 0.05 to 200 *Hz*, and then to a 10-order low-pass Chebyshev II filter with stopband ripple of 50 *dB* down and the stopband edge frequency of 49 *Hz*. For emphasizing the information enclosed in α and β rhythms extracted from EEG data, a 5-order band-pass Butterworth filter is further implemented for a bandwidth ranging from 8 to 30 *Hz*. Besides, an average reference is employed and the *Common Spatial Patterns* algorithm is carried out as a data-driven supervised decomposition of the EEG multi-channel data (He et al., 2012). All recordings are further digitized at 1000 *Hz* and down-sampled to supply the sampling frequency at 100 *Hz*. For each Motor Imagery (MI) class, the whole session was conducted without feedback, recording 100 repetitions per subject. Within the segment of interest lasting 4 *s* long, the subject was instructed to perform each MI task indicated by a pointing arrow on a screen. The segments lasting 2 *s* are interleaved with a blank screen and a fixation cross in the screen center.

**“*Klinik für Epileptology” Database* (KEDB)** (Andrzejak et al., 2001). This dataset, widely used in the automated epileptic seizure detection, consists of five subsets noted as A, B, C, D, and E. Each subset holds 100 single channel EEG segments lasting 23.6 *s*. Subsets A and B were acquired from five healthy subjects with eyes opened and closed, respectively. All signals from subsets C, D, and E came from five epileptic subjects. Subsets C and D included seizures-free interictal signals, which were measured on the epileptic zone and on the hemisphere opposite to the hippocampal formation of the brain. Set E contained epileptic signals recorded from each aforementioned location during an ictal seizure. Subsets C, D, and E were recorded intracranially. Besides, all provided EEG signals in KEDB were digitized at 173.61 *Hz* and 12 - bit resolution. To retain the relevant EEG information related to the studied normal and epileptic conditions, an average reference is used and all signals were filtered through a low-pass filter with a 40 *Hz* cutoff frequency. For the validation purpose, this data is tested on two problems with medical interest (Tzallas et al., 2009): *Bi-class* (2C), normal (A-type) and seizure (E-type) labeled recordings are distinguished; and *Three-class* (3C), closely represents the cases of actual medical applications, including three categories: normal (A-type EEG segments), seizure-free interictal (D-type EEG segments), and seizure (E-type EEG segments).

### 3.2. Extracted feature sets for identification of brain activity patterns

For either case of tested neural activity task (motor imagery or epileptic seizure detection), we validate the introduced EKRA approach in the following feature sets extracted from each data collection:

#### 3.2.1. Testing feature set extracted from MIDB

Let $\left\{Z_{n}\in {\mathbb{R}}^{C\times T}\right\}$ (*n*∈[1, *N*]) be a set of *N* EEG raw data trials for each subject, where *C*∈ℕ and *T*∈ℕ are the number of EEG channels and the amount of time samples, respectively. For discriminating the MI classes, we obtain a set of short-time features from each EEG trial ***Z***_*n*_ using the following extraction principles (Alvarez Meza et al., 2015):

–*Power Spectral Density-based parameters* (PSD): We estimate the PSD of each EEG channel based on the nonparametric Welch's method that calculates the widely known Fast Fourier Transform algorithm. The number of frequency bins is fixed according to the spectral band of interest [(4–30) *Hz*], where the most discriminative information for MI is concentrated (Rodríguez-Bermúdez et al., 2013). Due to the non-stationary nature of EEG data, a piecewise stationary analysis is carried out over a set of the extracted overlapping segments that are further windowed by a smooth time weighting window. Finally, the PSD includes the estimation of a modified periodogram vector from the Discrete Fourier Transform.
–*Hjorth-based parameters*: For each EEG channel, we compute such a representation from a set of the extracted overlapping segments. The set of Hjort parameters comprises *Activity* that is directly described by the signal power variance, *Mobility* that measures the signal mean frequency, and *Complexity* that estimates the frequency variations as the signal deviation from the sine shape (Arias-Mora et al., 2015).
–*Continuous Wavelet Transform* (CWT) parameters. Wavelet-based methods have been heavily exploited in MI research to capture the spectral dynamics of EEG trials that usually holds non-stationary spectral components. The CWT comprises an inner-product-based transformation that quantifies the similarity between a given time-series and a previously fixed base function, termed *mother wavelet*. Namely, the CWT-based parameters are extracted from each EEG channel by accomplishing their convolution with the scaled and shifted mother wavelet. We select the Morlet wavelet for the CWT analysis because its wave shape and EEG signals are alike and it allows extracting features better localized in the frequency domain (Aydemir and Kayikcioglu, 2011).
–*Discrete Wavelet Transform* (DWT) parameters. This principle adequately addresses the trade-off between time and frequency resolution in a non-stationary signal analysis. So, DWT provides multi-resolution and non-redundant representation by decomposing the considered time-series into some sub-bands at different scales, yielding more precise time-frequency information about the data. Aiming to extract the suitable time-frequency information from each EEG channel, we compute the detail coefficients as to include the (4–30) *Hz* band, resulting in the second and third levels of decomposition. Although there is a large selection of the available mother functions, we test the Symlet wavelet (Sym-7) that is closely associated with the electrical brain activity and proved to be appropriate in similar applications (Alomari et al., 2014).

Once we calculate all the short-time parameters, several of their statistical measures are further considered to extract the input feature matrix ***X***∈ℝ^*N*×*P*^. Namely, the mean, the variance, and the maximum values are estimated. Consequently, the row vector $x_{n}\in {\mathbb{R}}^{P}$ (*P* = *C* × *Q*) concatenates all features extracted from each *n*-th MI trial per channel, being *Q*∈ℕ the number of provided features. Thus, the total number of features per channel is 27 (6 for PSD, 9 – Hjort, 6 – CWT, and 6 for DWT). So, the size of the concatenated feature vector is *P* = 59 × 27, and the number of samples is *N* = 200.

#### 3.2.2. Testing feature set extracted from KEDB.

The rhythms carrying out clinical and physiological interest fall primarily within the following four spectral sub-bands: Delta denoted as δ with frequencies *f* < 4 *Hz*, Theta (θ, *f*∈[4, 8] *Hz*), Alpha (α, *f*∈[8, 13] *Hz*), and Beta rhythms (β, *f*∈[14, 30] *Hz*). Then, we select the linear filter bank for representation of EEG signals because they may be more accurately tuned to each rhythm frequency bandwidth. Therefore, we use five cepstral coefficients associated with δ, θ, α, and β rhythms, extracted as dynamic features as in Duque-Muñoz et al. (2014). As a result, instead of a widely used scalar-valued parameter set extracted from the EEG signal, the neural activity relating to epileptic seizures is detected by using a vector set of short-time rhythms.

All the baseline algorithms required to compute the features were developed based on the Signal Processing toolbox of Matlab 2013b. Note that we perform validation on two different training sets, intending to test the EKRA approach under very diverse neural activity data, namely, motor Imagery discrimination and epileptic seizure detection. Both sets are multiclass and cover all the brain regions. Besides, the variability of the former data collection is very broad, while the latter data hold more concentrated dynamics along the time. This aspect is important to test since EKRA benefits from the information about the variability of the input space. Another regard to considering is that either database is publicly available, and widely used on state-of-the-art literature, making possible to compare their performance with other used approaches of training.

### 3.3. Validation of the enhanced kernel-based relevance analysis

With the purpose of assessing the influence of each one of the computation stages explained above, we conduct validation of the proposed EKRA method for the following two training scenarios of identification of brain activity patterns:

(i) The EKRA *Feature selection* method (noted as *EKRA–S*) provides a better understanding of the input feature set, and thus, facilitates the physiological interpretation task. To this end, all relevance features are sorted in decreasing order of the achieved contribution amplitude, yielding the ranking EKRA vector ϱ. Therefore, supplying to the validated classifier one-by-one the ranked features, the accuracy dependence is performed through a nested 10-fold cross-validation scheme, according to the ranked, relevant features. It is worth noting that the use of the ranking vector avoids the inclusion of any heuristic nor greedy feature selection search (like the conventional forward-backward approaches), demanding huge computational burden. For comparison regarding the physiological interpretation, the proposed EKRA is contrasted with a baseline *Variance-based Relevance Analysis* (VRA) that ranks the short-time input features grounded on a variability criterion. Namely, VRA computes a relevance vector based on a linear transformation of the input representation (Daza-Santacoloma et al., 2009).

(ii) The EKRA *Enhanced feature selection* (*EKRA–ES*) incorporates the projection to the EKRA–S procedure, aiming to raise the performed accuracy of brain activity identification (see section 2.2). So, a mapped feature set is estimated to encode more accurately the available discriminant neural patterns through the embedding matrix ***Y*** as explained above.

For EKRA calculation in Appendix A in Supplementary Material, the free parameters are fixed as follows: The initial guess ***A***^0^ is adjusted according to the well-known principal component analysis approach, σ^0^ is fixed to the median of the input data Euclidean distances, the gradient descent tolerance is set to 1*e* − 6, the maximum number of iterations is empirically limited up to 300, and the number of relevant dimensions (*M*_*S*_ and *M*_*E*_) are adjusted as to hold 95% of the variance explained. Note that *EKRA* learns two projection matrices (***A*** and ***A***′), resulting in a computational complexity of $O\left(N^{2}\right)$. However, this procedure is carried out off-line. The EKRA–S and EKRA–ES Matlab implementation codes are publicly available^2^.

In each task at hand, we primarily perform a visual inspection as the simplest way of representing, graphically, the dependence of the classification performance from the relevant features, taking into consideration the applied feature extraction principle as well as the measuring EEG channel or brain region. Besides, the estimation of the classification performance is also considered. In either scenario of validation, a k-nearest neighbor classifier is employed to identify the brain patterns under stimuli/conditions, for which the number of nearest neighbors is tuned within the range {1, 3, 5, 7, 9, 11} based on the system accuracy achieved, operating a nested 10-fold cross-validation to avoid any over-fitting results.

## 4. Results and discussion

### 4.1. Validation results on motor imagery tasks

#### 4.1.1. Relevance analysis performed by the feature selection scenario

**Figure 1:**
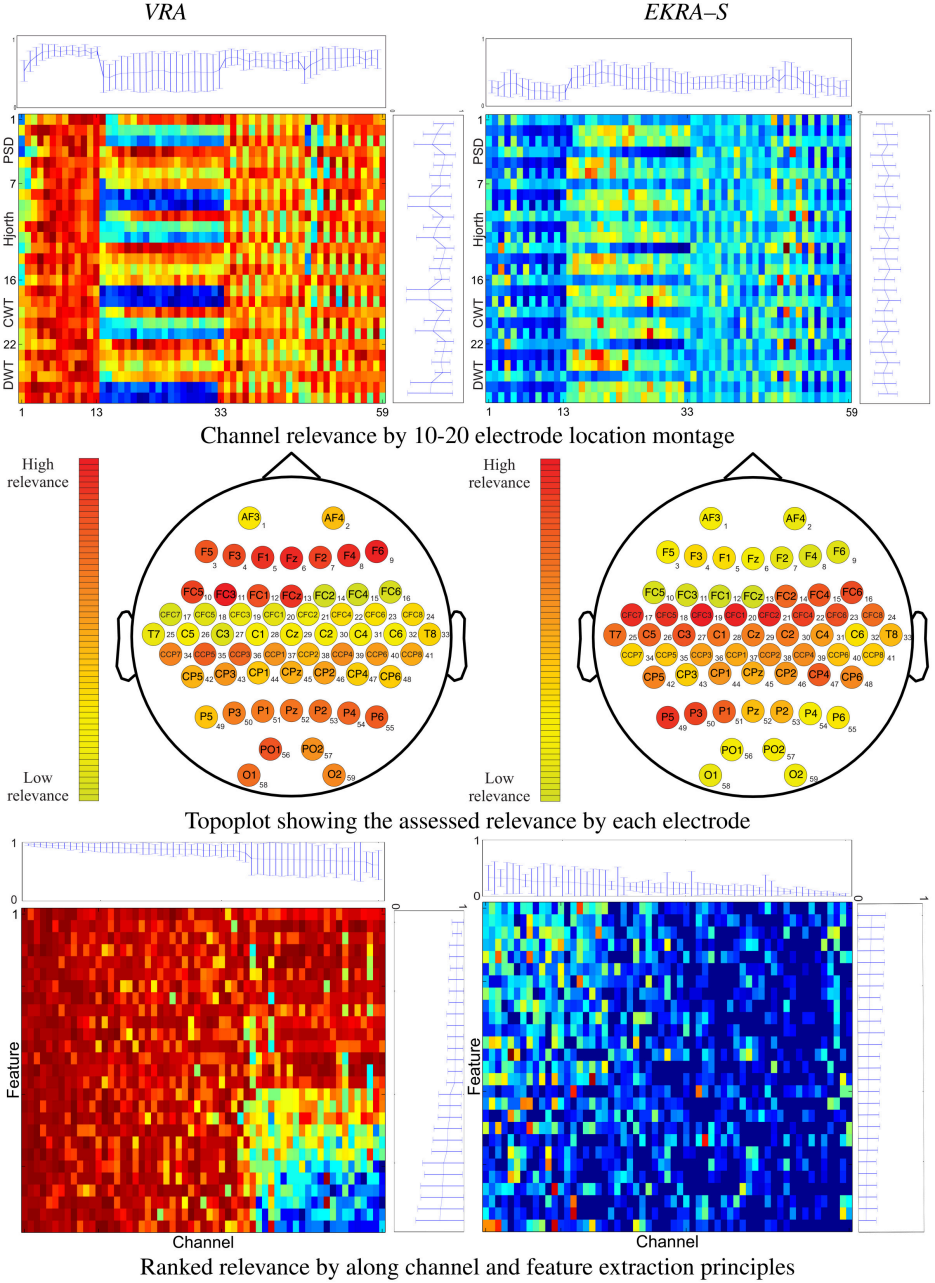
2D representation (top and bottom rows) and topoplots (middle row) of the performed relevance analysis on motor imagery task. The plot at the top shows the marginal relevance (mean and standard deviation) per channel. The topoplots are computed by averaging the input feature relevance values in ϱ regarding the EEG montage (channels). The right-side plot shows the marginal relevance averaged for all considered principles of feature extraction.

We initially consider the performed relevance analysis for the feature selection as shown in Figure 1 that displays the estimated relevance averaged through all tested subjects. In each 2D representation, the abscissa indicates the cardinal assigned to each one of 27 features, while the horizontal axis represents the cardinal of the 59 channels labeled by the international 10–20 electrode location montage. The proposed subspace-based methodology rests on measuring the covariance-based evolution of underlying time-varying signals, providing the best discrimination among the classes. To this end, EKRA quantifies the short-time relevance, using the centralized kernel alignment that is adjusted to learning the linear projection that best discriminates a given input feature space, assessing the similarity between the projected input data and stimulus/condition labels. For the sake of comparison, validation also comprises the baseline variance-based relevance analysis (VAR) that only estimates the time-varying feature contribution in the original input space over time, but regarding the unsupervised classifier performance. Although the validation performed on two data collections shows that the obtained preliminary results are encouraging, some additional aspects should be further considered:

The performed relevance by the VRA algorithm (left column) allows to appraise the contribution from every single electrodeposition, resulting in three channel groups of relevance that can be spatially identified, namely, the channels numbered as #1–13, #14–33, and #34–59. To quantify the contribution, we calculate the marginal relevance per channel averaged through all involved features. Thus, the first labeled 13 channels (placed over the association cortex) perform the strongest contribution, having even the lowest dispersion as can be seen in the top plot of Figure 1. A lower relevance value is supplied by the electrode positions that collect the neural activity in the anterior parts of the anterior parietal cortex (#33–59). The lowest relevance (having even the highest variance) is produced by the electrode positions that relate to the precentral gyrus (#14–28) and the dorsal lateral premotor area (#30–33). In the case of EKRA–S, the three spatially distinct groups remain, but the estimated relevance per channel is different from the one assessed by VRA. As seen in the top plot of Figure 1, the electrode positions labeled as #14– 32 now become the most important succeeded by the group #33– 59. In contrast to the VRA approach, the channels placed over the association cortex (#1–13) perform the worst relevance. As regards the relevance assessed by each principle of feature extraction, the tested feature sets do not cluster so distinctly for either used selection algorithm, though most of the characteristics behave differently depending on the electrode position (see right-side plots of Figure 1).

With the aim of further exploring the spatial distribution of the carried out feature selection, all computed relevance values are arranged in the 10–20 channel montage as displayed in the topoplots of the middle row of Figure 1. It is worth noting that we describe the MI brain activity performed by a hypothetical medium person due to the estimated relevance planes are averaged across all subjects. So, VRA produces the highest contribution of relevance for the middle frontal gyrus that is represented by channels F5, F3, F4, and F6. However, the middle frontal gyrus should not be related to any imagery stimulation (Hanakawa et al., 2003). Rather, this brain area activates as a response to body movements, e.g., powerful EEG artifacts. In other words, the presence of EEG channels with high-energy disturbances may mislead the VRA estimator, identifying the MI patterns wrongly. Instead, EKRA–S assigns the bigger values of relevance to the EEG channels placed over two brain areas that indeed are commonly related to MI tasks. Namely, the posterior superior parietal cortex (P3, P1, PZ, and P2), and the left precentral sulcus at the level of the middle frontal gyrus (CFC5, C3, CFC3, C1, and CFC1). Furthermore, the middle frontal gyrus has the lowest contribution, weakening the influence of movement artifacts. To better visualize the joint channel-feature relationship, we rearrange each 2D representation so that the relevance estimates are now ranked in decreasing order along the channel and feature extraction principle axes. Importantly, all features that do not contribute to 95% of the variance explained are zero-valued. Comparison of 2D representations of the bottom row in Figure 1 allows concluding that each employed relevance estimator associates the input training set differently, playing a very significant role in appraising the channel-feature contribution.

Therefore, the baseline VRA algorithm produces higher values of the relevance marginal (see the top and right-side plots of each 2D representation) in comparison to the proposed EKRA–S, suggesting that the latter approach encodes the whole brain activity task into a lower number of features. The following two facts can explain this advantage of EKRA–S: (i) the use of the MI label information to reveal features, which must be salient regarding the studied paradigm. Thus, the brain activity patterns are better localized. (ii) Representation through enhanced RKHS allows dealing with complex neighboring data dependencies, rejecting more efficiently redundant features and highlighting coherent spatial regions (EEG channels) regarding the studied MI paradigm. In contrast, VRA mainly explains the relevance concerning its energy-based cost functional that emphasizes the brain regions with intense activity, which are activated during the time the stimuli goes. Yet, this assumption does not necessarily hold for MI tasks.

**Figure 2:**
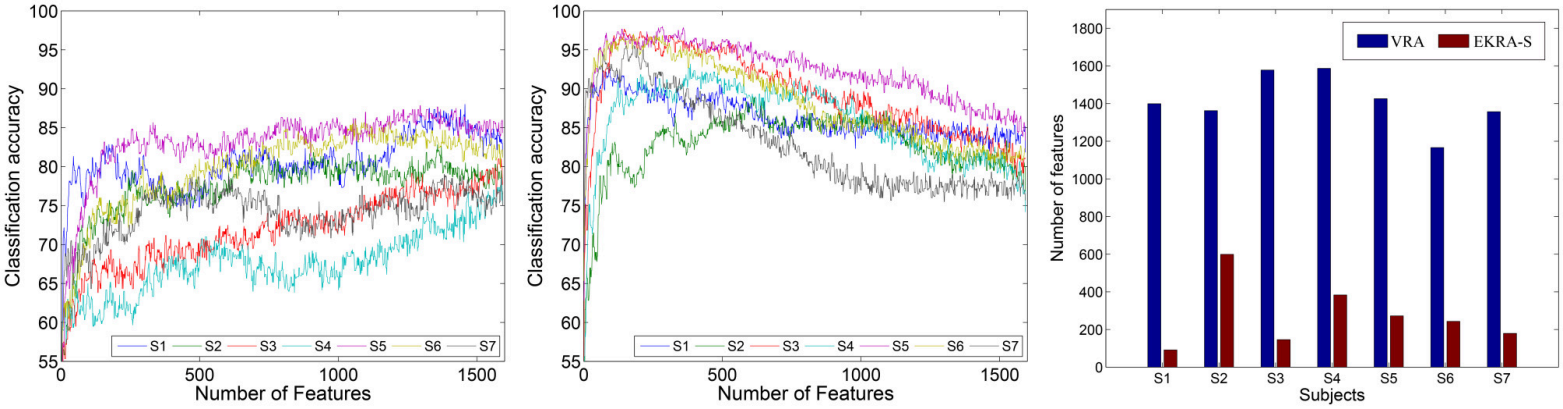
Motor Imagery discrimination accuracy performed by the feature selection strategy. The achieved average accuracy of classification is computed using a nested cross-validation approach, adding one by one the features ranked. VRA, left column; EKRA-S, middle column; DR, right column. Results are displayed concerning each studied subject.

With the aim of estimating the classification performance of the contemplated MI tasks, we assume the selected training set as the one containing the minimum amount of features to reach the maximum classification accuracy. To this end, the *k*-nearest neighbor classifier is fed by adding one by one the relevant features, which have been previously ranked in decreasing order. In average for all subjects, VRA performs an accuracy close to ~85.16 ± 3.88% and clearly falls behind the EKRA–S algorithm that reaches ~95.71 ± 3.01% as seen in Figure 2. Moreover, the number of selected training features is also shown for each subject. Regardless of the used feature selection strategy, the performed accuracy has some fluctuations due to the inter-subject variability that has been already reported for spatial patterns and spectro-temporal characteristics of brain signals in Motor Imagery tasks (Blankertz et al., 2007). One more reason causing the performance fluctuations is the simplicity of the employed *knn* classifier. Therefore, the use more elaborate classifiers (like SVM) should deal better with the fluctuations. Along with the MI discrimination performance, another important aspect to explain is the number of selected training features of the whole input set (1593). As displayed, VRA chooses about 1, 410 features, but EKRA–S does only 275 features. Consequently, a dimension reduction is close to one and 5.8, respectively, averaged for all subjects. Therefore, EKRA–S is as much as five times more efficient than the contrasted baseline estimator, regarding the reduction dimension processing.

**Figure 3:**
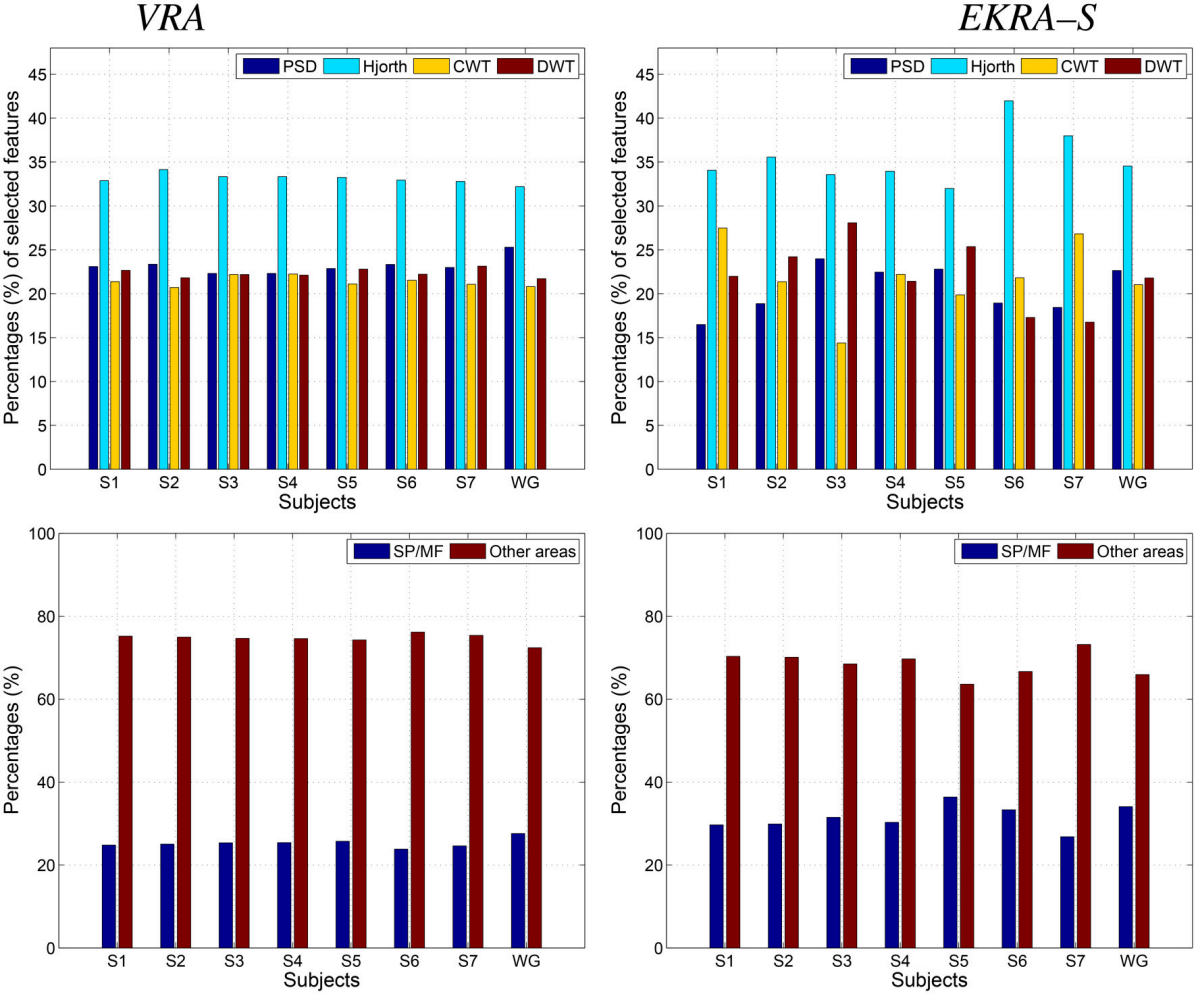
Contribution of the selected feature set to the Motor Imagery discrimination performance. VRA, left column; EKRA-S, right column Feature extraction principle (PSD, Hjorth, CWT, and DWT parameters) - top row, Brain area (SP and MF) - bottom row. Results are displayed concerning each studied subject.

For both selected feature sets, a detailed analysis results in the following findings:

– Apparently, the Hjorth principle of extraction supplies the features with the highest relevance, contributing the most to the discrimination of MI classes regardless of the used relevance estimator. The remaining spectral characteristics have a comparable contribution, though some differences apply for EKRA–S.
– Regarding the proportion of features encoding MI information (the *superior parietal* plus *middle frontal gyrus*), EKRA–S produces a higher number of salient features.
– As one of the biggest challenges in BCI research, it is worth mentioning the inter-subject variability of spatial patterns and spectro-temporal characteristics of brain signals (Blankertz et al., 2007). In the contemplated MI task, some subjects might not focus their gaze in the proper direction, and thus, the EEG recordings will not be reliable for meaningful interpretation. From the comparison plots of relevance in Figure 3, it follows that EKRA–S better adapts the BCI system for each particular subject, at least, in terms of revealing the most discriminating features. Also, the layout of Figure 1 is enhanced by including a circular representation that embraces the subject variability estimated for each channel, pointing out on the places where the proposed technique captures better the individual patterns.
– Either relevance approach (VRA or EKRA–S) provides a high variability among subjects, mainly focusing on two brain areas: Posterior parietal cortex (SP) and left precentral sulcus at the level of the middle frontal gyrus (MF). Note that both areas are commonly related to MI tasks. However, the relevance, given by EKRA–S to brain areas related to MI tasks (SP and MF), is higher in each subject than the relevance assigned by the VRA approach. In other words, though the variability among subjects is similar in both methods, EKRA–S enhances the relevance of brain areas that are more related to MI tasks.

#### 4.1.2. Relevance analysis performed in enhanced feature selection scenario:

In this case, we calculate the performance of the proposed relevance analysis for both cases of consideration: feature selection (EKRA-S) and enhanced feature selection (EKRA-ES). Results are given regarding the classifier accuracy achieved for the contemplated MI task for every subject. In the former case, our proposal reaches an averaged accuracy 95.71±03.01 and 96.71±01.84, respectively, outperforming the contrasted baseline VRA that produces 92.86±03.77. For the sake of comparison, we also include the accuracy estimated by the approach submitted in Zhang et al. (2012) that selects an extracted spatio-temporal feature set, from which a non-linear regression for predicting the time-series of class labels is applied. Another compared work is the one in (He et al., 2012) that uses an adaptive frequency band selection of the spatial preprocessed features that feed an SVM classifier. Lastly, we consider the approach in Higashi and Tanaka (2013) involving common space-time-frequency patterns to design the time-windows used for the MI task. As seen in Table 1, all referred training strategies underperform the proposed relevance analysis method.

**Table 1:** Performed classification accuracy for Motor Imagery discrimination (average ± standard deviation [%]).

**Subject**	**VRA**	**EKRA-S**	**He et al., 2012**	**Zhang et al., 2012**	**Higashi and Tanaka, 2013**	**EKRA-ES**
# 1	91.50 ± 05.29	94.16 ± 05.30	67.70 ± 02.20	77.20 ± 00.03	92.30 ± 02.50	**98.00** ± **02.58**
# 2	**96.50** ± **03.37**	90.16 ± 05.88	70.70 ± 01.20	70.80 ± 00.02	90.60 ± 7.20	93.00 ± 05.37
# 3	91.50 ± 04.74	**98.50** ± **08.57**	83.90 ± 01.30	–	–	97.50 ± 03.54
# 4	87.00 ± 06.32	94.50 ± 07.01	93.00 ± 01.20	–	–	**96.50** ± **04.12**
# 5	91.50 ± 07.47	**98.50** ± **04.60**	93.20 ±01.20	–	–	97.50 ± 03.54
# 6	98.50 ± 02.42	97.66 ± 04.82	–	76.80 ± 00.03	93.30 ± 03.60	**98.50** ± **00.01**
# 7	93.50 ± 07.09	**96.50** ± **03.45**	–	80.00 ± 00.03	94.10 ± 04.10	96.00 ± 03.16
**Mean**	92.86 ± 03.77	95.71 ± 03.01	81.70 ± 12.06	76.20 ± 03.87	92.58 ± 01.51	**96.71** ± **01.84**

*Notation (-) stands for Not provided. Note that the accuracy of EKRA-S, EKRA-ES, and VRA is estimated as the highest value performed for each tested subject under a nested cross-validation scheme. Bold values indicate the best results*.

### 4.2. Validation results on epileptic seizure detection

#### 4.2.1. Relevance analysis performed in the feature selection scenario:

We test both, VRA and EKRA-S, approaches as a feature selection tool of the spectral coefficients extracted from the physiological rhythms (δ, θ, α, and β). Since the KEDB dataset only has one-channel EEG recordings, the physiological interpretation of the selected feature set only covers the influence of the physiological waveforms on the two possible challenges of epileptic seizure detection. The selected feature set is calculated as in the motor imagery task for which the accuracy of the *k*-nearest neighbor classifier is also performed through the nested 10-fold cross-validation scheme.

**Figure 4:**
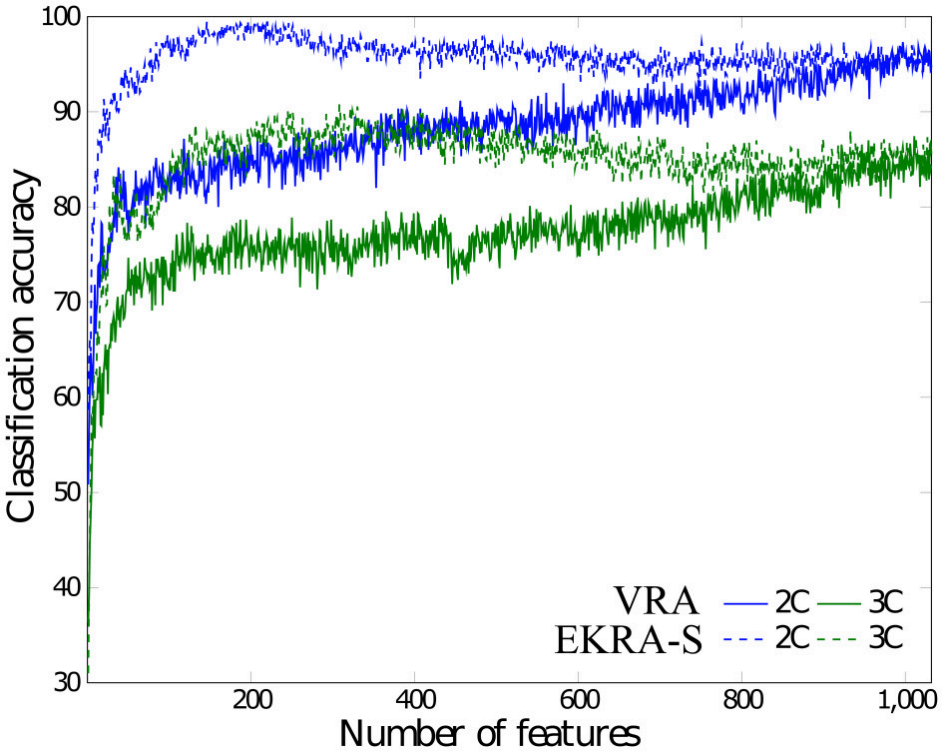
Performed accuracy for epileptic seizure detection using each compared approach of feature selection. The achieved average accuracy of classification is computed using a nested cross-validation approach, adding one by one the features ranked. VRA (continuous lines), EKRA–S (dashed lines). The blue and green colors hold for the 2C and the 3C problem, respectively.

As seen in Figure 4, either comparative approach of feature selection attains the highest accuracy (100%) for the bi-class task. Further, EKRA-S betters the baseline VRA for the tasks of three classes (96.00 vs. 90.78%, respectively). Regarding the number of selected training features, once again the EKRA-S approach outperforms VRA in all tasks. Note that the VRA classification accuracy increases as the number of features grow, requiring the full input feature set to reach the maximum performance. Meanwhile, the addition of more features drops the performance once the EKRA-S approach gets the highest accuracy, indicating that the inclusion of other features may be redundant. As a result, the dimension reduction is two and three times bigger than the one obtained by VRA for the 2C and 3C tasks, respectively. This aspect can be of benefit for reliable online monitoring of traces of interictal/ictal states of epilepsy since the demanded time-window of EEG analysis may be remarkably shortened.

**Figure 5:**
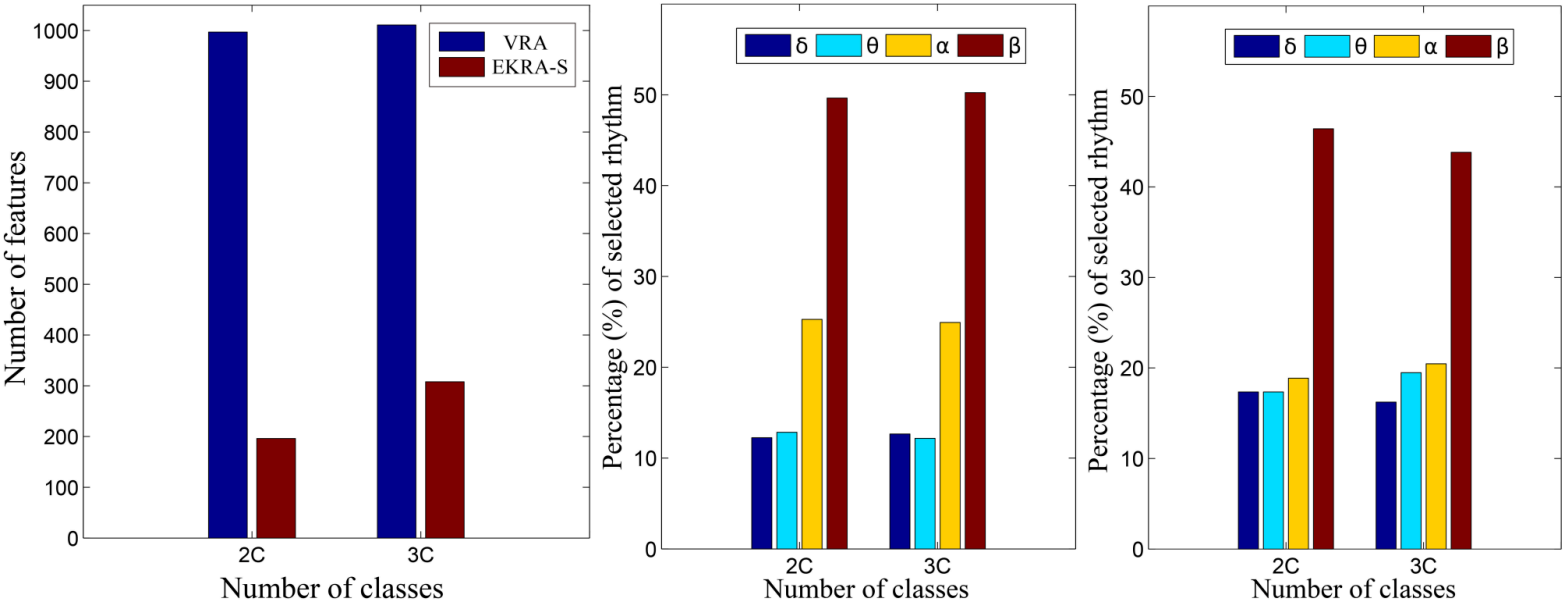
Relevant rhythms regarding the seizure detection tasks. DR - left column. The number of selected features is shown for each provided classification problem (2C and 3C), in blue and red for VRA and EKRA, respectively. VRA-based rhythms selection - middle column, EKRA-S-based rhythms selection - right column. The percentage of selected rhythms are shown in colors for the 2C and the 3C problems.

Figure 5 shows the normalized relevance values that are estimated for each rhythm. By the VRA estimator, the selected features make α and β waveforms the most relevant for all considered tasks. At the same time, low-frequency rhythms (δ, θ) exhibit modest values of relevance. Although EKRA-S infers a similar contribution of the rhythms, the relationship between the high to low-frequency rhythms decreases as the number of classes increases. This result indicates on the energy redistribution, taking place as the complexity of the task increases as has been explained in similar works (Duque Muñoz et al., 2015).

For the sake of comparison, both proposed strategies for relevance analysis (EKRA-S and EKRA-ES) are contrasted with some recent approaches for epileptic seizure detection. Although this comparison may not be entirely fair due to different details on the testing procedures (Kumar et al., 2010; Zandi et al., 2010), it seems to be the best possible option. The best classification accuracy achieved by each contrasted approach of epileptic seizure detection is displayed in Table 2, showing that almost all benchmarked approaches provide a high classification accuracy that ranges from 99.5% to 100% for a *Bi-class* problem, and from 90.78 to 100% for a *Three-class* task. Note that we employ a nested 10-fold cross-validation to avoid any over-fitting of the discrimination system.

**Table 2:** Accomplished classification results for epileptic seizure detection.

**2-class**			**3-class**		
**Authors**	**Features/Classifier**	**Accuracy**	**Authors**	**Features/Classifier**	**Accuracy**
Srinivasan et al., 2005	*t-f* analysis/RNN	99.6	Ghosh-Dastidar et al., 2008	PCA-RBF/ANN	96.60
Gandhi et al., 2011	WT/PNN	99.99	Naghsh-Nilchi and Aghashahi, 2010	EV/MLP NN	97.50
Polat and Gunes, 2007	PCA FFT/AIRS	100	Tang and Durand, 2012	PSD+CLZ/SVMA	98.72
Zafer et al., 2011	CC+PSD/vot. rule	100	Martínez-Vargas et al., 2012	TFR-2DPCA/*k-nn*	98.80
Martínez-Vargas et al., 2012	TFR-2DPCA/*k-nn*	100	Tzallas et al., 2009	**t*−*f** analysis/ANN	100
Tzallas et al., 2009	*t-f* analysis/ANN	100	Duque-Muñoz et al., 2014	short-time/*k-nn*	100
Duque-Muñoz et al., 2014	short-time/*k-nn*	99.5	*Proposal*	EKRA-S	**90.78**
*Proposal*	EKRA-S	**100**	*Proposal*	EKRA-ES	**96.00**
**Proposal**	EKRA-ES	**100**			

*The EKRA-S and EKRA-ES approaches are compared against state-of-the-art methods concerning the average classification accuracy [%]. Bold values indicate the best results*.

Another aspect to reflect is the influence of the parameter tuning. As seen in Table 3 that shows the confidence of the point classification estimates provided by the EKRA free meta-parameter, there are small fluctuations in performance among the training folds, which are calculated by the nested cross-validation strategy to avoid any overestimation effect of the performed accuracy. Hence, the proposed approach together with its optimization strategy allows extracting relevant brain patterns, providing a solid classification accuracy.

**Table 3:** EKRA free parameters values for each provided brain activity task. The average ± the standard deviation are presented.

**Brain activity task**		***M*_*S*_**	***M*_*E*_**	***k*-neighbors**	**σ**
Motor imagery	S1	796.00 ± 0.00	94.70 ± 1.64	4.20 ± 1.93	0.91 ± 0.00
	S2	796.00 ± 0.00	108.90 ± 0.32	3.20 ± 0.63	0.91 ± 0.01
	S3	796.00 ± 0.00	122.10 ± 0.32	3.40 ± 0.84	0.91 ± 0.00
	S4	796.00 ± 0.00	116.90 ± 0.32	3.40 ± 1.26	0.93 ± 0.00
	S5	796.00 ± 0.00	117.80 ± 0.42	3.20 ± 0.63	0.93 ± 0.00
	S6	796.00 ± 0.00	104.20 ± 0.63	3.00 ± 0.00	0.92 ± 0.01
	S7	796.00 ± 0.00	90.90 ± 1.45	3.80 ± 1.93	0.86 ± 0.01
Epileptic seizure detection	2-Class	516.00 ± 0.00	111.90 ± 0.74	3.00 ± 0.00	1.07 ± 0.02
	3-Class	516.00 ± 0.00	143.50 ± 0.85	4.20 ± 2.15	1.10 ± 0.00

*The number of selected and projected features in EKRA (M_S_ and M_E_), as well as the number of neighbors and the Gaussian kernel bandwidth in the k-nearest neighbor classifier (k and σ), are studied according to a nested cross-validation scheme*.

## 5. Conclusions

We discuss a novel kernel-based approach for the feature relevance analysis to enhance the automatic identification of brain activity patterns. To this end, the proposed relevance analysis incorporates two kernel functions to take advantage of the available joint information associating neural responses to an individual stimulus/conditions with the corresponding labels. Then, kernel alignment learns all relevant patterns from the short-time input features. Validation of the proposed Kernel-based Relevance Analysis is carried out in two scenarios of training: feature selection and enhanced feature selection. In particular, two tasks of brain activity identification are studied that exhibit highly non-stationary behavior: motor imagery discrimination and epileptic seizure detection.

With the aim to encode two different notions of similarity, the need for handling a couple of kernels encourages the use of the well-known kernel alignment to unify both tasks into a single optimization framework. Nonetheless, the selection of distances, which implement each aligned kernel as well as the same alignment, mostly determines the effectiveness of the kernel-based approach for a given application. In the particular case of brain activity identification, we rely on the Mahalanobis distance to carry out the pairwise comparison between samples based on the Gaussian kernel. Thus, a linear projection is further learned from the employed CKA-based functional as an alternative to highlight the salient input features, taking advantage of the non-linear notion of similarity behind the selected kernels. For the sake of simplicity, the iterative gradient descent optimization is employed to calculate the projection matrix and the Gaussian kernel free parameter.

To implement EKRA as a feature selection tool – (EKRA-S), we introduce a feature relevance vector index devoted to measuring the contribution of each one of the input features in building the projecting CKA matrix. So, we assess the selected feature set that satisfies a given stopping criteria (namely, we fix the proportion of variance explained) by ranking this contribution. Thus, the feature selection using EKRA-S demands small feature sets with the benefit of providing a better interpretation of the space brain activity distribution and the principle of employed feature extraction. Besides, the EKRA-based ranking separates redundant features, which usually tend to drop the system accuracy. As another advantage, the EKRA-S approach adapts the relevance analysis to include the inter-subject variability. This aspect remains one of the most challenging issues of training for BCI systems. With the purpose to improve interpretation on a neurophysiological basis, the proposed Kernel-based Relevance Analysis is designed to take advantage of the measured brain activity, associating neural responses to a given stimulus condition and aiming to assess the contribution of each spatial electrode location to the identification performance. As a result, the EKRA-based relevance mainly highlights those regions that are indeed neurophysiologically related to the Motor imagery tasks with the benefit of providing a confident and competitive accuracy performance. So, the EKRA enhances the interpretability of brain activity patterns, enabling to discuss, verify and even improve the performed results. Therefore, EKRA as a feature selection tool can reach a suitable classification accuracy with a high dimension reduction factor, providing better physiological interpretation of the brain activity patterns.

In the other training scenario of enhanced feature selection (EKRA-ES), we use the relevance index vector to estimate the representation space that optimizes a trade-off between separability and no redundancy of the available neural patterns. As a result, our proposal outperforms those compared approaches that carry out the multivariate feature selection and/or dimension reduction. Indeed, the EKRA-based enhanced feature spaces handle the brain activity complexity to support further classification stages regarding system accuracy and reliability.

Regarding the EKRA shortcomings, we must clarify that its current implementation, based on gradient descent optimization, requires a considerable computational load of a large number of samples (thousands). Besides, EKRA-based relevance analysis could be biased under imbalanced data scenarios, due to the CKA-based cost function tends to accentuate data relations within the class with the highest number of samples.

As the future work, the authors plan to improve the EKRA algorithm by introducing more elaborate alignment functions and different kernel mappings with the aim to get a better description of non-stationary signals, which can be immersed in either Gaussian or non-Gaussian noise conditions. For example, the measures based on information theory would be of benefit (Giraldo et al., 2015). Besides, hidden inter-channel relationships should be estimated to enhance the extraction of brain activity patterns within the EKRA-based framework (Dauwan et al., 2016). Alike, a more elaborate study regarding the EKRA optimization process must be carried out by including second derivatives and low-rank approximations of the kernel matrices to favor its convergence, and weighting approaches to deal with imbalanced tasks (Jian et al., 2016). Furthermore, the EKRA benefits should be studied regarding the EEG reference used, e.g., the average reference vs. the Reference Electrode Standardization Technique; in particular, if we intend to extend our proposal to other salient brain activity applications (Yao, 2001; Chella et al., 2016; Yao, 2017).

## Author contributions

AA: EKRA theoretical development and coding. Motor imagery database tests and analysis. Support the manuscript writing. AO: Epileptic database tests and analysis. Support the manuscript writing. GC: EKRA theoretical development, BCI database analysis, and support the manuscript writing.

### Conflict of interest statement

The authors declare that the research was conducted in the absence of any commercial or financial relationships that could be construed as a potential conflict of interest.

## Footnotes

^1^http://bbci.de/competition/iv/desc_1.html. Dataset 1 used in the BCI competition IV 2008

^2^https://github.com/andresmarino07utp/EKRA-ES
