# Supplementary material for: Kernel-Based Relevance Analysis with Enhanced Interpretability for Detection of Brain Activity Patterns
Source: Front Neurosci. 2017 Oct 6;11:550. doi: 10.3389/fnins.2017.00550 (PMC5635061; doi:10.3389/fnins.2017.00550)
Supplement: Supplementary file 1 [file DataSheet1.docx]

APPENDIX A EKRA CALCULATION BASED ON GRADIENT-DESCENT OPTIMIZATION

In the empirical estimation of Eq. (4), the rotation matrix relies on the explicit objective function as follows (Brockmeier et al., 2014):

$$\hat{\rho}\left(\boldsymbol{K_X}(\boldsymbol{A},\sigma),\boldsymbol{K_l}\right)=\log\left(\operatorname{tr}\left(\boldsymbol{K_X}(\boldsymbol{A},\sigma)\tilde{\boldsymbol{I}}\boldsymbol{K_l}\tilde{\boldsymbol{I}}\right)\right)$$
$$-0.5\log\left(\operatorname{tr}\left(\boldsymbol{K_X}(\boldsymbol{A},\sigma)\tilde{\boldsymbol{I}}\boldsymbol{K_X}(\boldsymbol{A},\sigma)\tilde{\boldsymbol{I}}\right)\right)+\rho_0,$$

where $\rho_0 \in \mathbb{R}$ is a certain constant that is not dependent on $\boldsymbol{A}$.

Apart from learning the optimal projection matrix (noted as $\hat{\boldsymbol{A}}$), the optimizing approach in Eq. (6) also demands tuning of the kernel bandwidth σ. To deal with this joint parameter estimation, we propose to optimize one variable recursively at a time while the other variable remains unchanged. With this purpose, we apply a gradient descent approach to solving the optimizing EKRA task iteratively. Thus, under a fixed σ, the objective function gradient in Eq. (8) with regard to the maximizing parameter $\boldsymbol{A}$ results in the form:

$$\nabla_{\boldsymbol{A}}\left(\hat{\rho}\left(\boldsymbol{K_X}(\boldsymbol{A},\sigma),\boldsymbol{K_l}\right)\right)=-4\boldsymbol{X}^\top\left(\left(\boldsymbol{G}\circ\boldsymbol{K_X}(\boldsymbol{A},\sigma)\right)\right.$$
$$\left.-\operatorname{diag}\left(\boldsymbol{1}^\top\left(\boldsymbol{G}\circ\boldsymbol{K_X}(\boldsymbol{A},\sigma)\right)\right)\right)\boldsymbol{X}\boldsymbol{A},$$

where notations $\operatorname{diag}(\cdot)$ and $\circ$ denote the diagonal operator and Hadamard product, respectively. $\boldsymbol{G}\in\mathbb{R}^{N\times N}$ is the objective function gradient expressed in terms of $\boldsymbol{K_X}(\boldsymbol{A},\sigma)$, and calculated as follows:

$$\boldsymbol{G}=\nabla_{\boldsymbol{K_X}(\boldsymbol{A},\sigma)}\left(\hat{\rho}\left(\boldsymbol{K_X}(\boldsymbol{A},\sigma),\boldsymbol{K_l}\right)\right)$$
$$=\frac{\tilde{\boldsymbol{I}}\boldsymbol{K}_l\tilde{\boldsymbol{I}}}{\operatorname{tr}\left(\boldsymbol{K_X}(\boldsymbol{A},\sigma)\tilde{\boldsymbol{I}}\boldsymbol{K}_l\tilde{\boldsymbol{I}}\right)}-\frac{\tilde{\boldsymbol{I}}\boldsymbol{K_X}(\boldsymbol{A},\sigma)\tilde{\boldsymbol{I}}}{\operatorname{tr}\left(\boldsymbol{K_X}(\boldsymbol{A},\sigma)\tilde{\boldsymbol{I}}\boldsymbol{K_X}(\boldsymbol{A},\sigma)\tilde{\boldsymbol{I}}\right)}$$

Provided the initial guess $\boldsymbol{A}^o$, we renew the estimation for $\boldsymbol{A}$ by applying the following gradient-descent update rule:

$$\boldsymbol{A}^{t+1}=\boldsymbol{A}^t-\mu_A^t\nabla_{\boldsymbol{A}^t}\left(\hat{\rho}\left(\boldsymbol{K_X}(\boldsymbol{A}^t,\sigma^t),\boldsymbol{K_l}\right)\right)$$

where $\mu_A^t\in\mathbb{R}^+$ is the step size of the learning rule, and $\boldsymbol{A}^t$ and σ^t are the samples we use at the time step t.

Due to the kernel bandwidth allows scaling all pairwise distances on the projected space $\boldsymbol{Y}^t{=}\boldsymbol{X}\boldsymbol{A}^t$, we estimate σ^t within the framework of *Information Theoretic Learning*, intending to highlight the most contributing similarities between samples (?). Specifically, we maximize the overall variability of the information potential computed for all samples over $\boldsymbol{Y}^t$ in terms of the kernel bandwidth parameter so that all information force magnitudes spread more widely. That is:

$$\sigma^t=\arg\max_{\sigma}\operatorname{var}\left\{v_\xi\left(\boldsymbol{y}_n^t|\sigma\right):\forall\boldsymbol{y}_n^t\in\boldsymbol{Y}^t\right\},$$

where $\mathrm{var}\left\{v_\xi\left(\boldsymbol{y}_n^t|\sigma\right)\right\}=\mathbb{E}_{\boldsymbol{y}_n^t}\left\{\left(v_\xi\left(\boldsymbol{y}_n^t|\sigma\right)-\tilde{v}_\xi\left(\boldsymbol{Y}^t|\sigma\right)\right)^2\right\}$, $\tilde{v}_\xi\in\mathbb{R}$ is the ξ-order *Information Potential* (IP) of $\boldsymbol{Y}^t$ that is defined as $\tilde{v}_\xi(\boldsymbol{Y}^t|\sigma)=\mathbb{E}_{\boldsymbol{y}_n^t}\left\{v_\xi\left(\boldsymbol{y}_n^t|\sigma\right)\right\}$, and $v_\xi\left(\boldsymbol{y}_n^t|\sigma\right)$ is the IP of each sample $\boldsymbol{y}_n^t$ computed as below:

$$v_\xi\left(\boldsymbol{y}_n^t|\sigma\right)=\frac{1}{N^{\xi-1}}\sum_{n'=1}^{N}\left(\kappa_X\left(\boldsymbol{x}_n\boldsymbol{A}^t,x_{n'}\boldsymbol{A}^t;\sigma\right)\right)^{\xi-1},$$

where $\mathrm{d}^2\left(\boldsymbol{x}_n\boldsymbol{A}^t,\boldsymbol{x}_{n'}\boldsymbol{A}^t\right)=\|\boldsymbol{y}_n^t-\boldsymbol{y}_{n'}^t\|_2^2$ is calculated for κ_X as the L_2-norm distance.

Therefore, under a fixed $\boldsymbol{A}^t$, the gradient descent approach can be applied to solve Eq. (12). To this end, we use the *Renyi's Quadratic Entropy* (i.e., ξ=2), yielding the following derivative in terms of σ:

$$\frac{d}{d\sigma}\mathrm{var}\left\{v_2\left(\boldsymbol{y}_n^t|\sigma\right)\right\}=\Big(\left(\sigma\right)^2\mathbb{E}_{nn'}\left\{\chi^2\left(\boldsymbol{y}_n^t|\boldsymbol{y}_{n'}^t\right)\right\}$$
$$-\tilde{v}_2\left(\boldsymbol{Y}^t|\sigma\right)\mathbb{E}_{nn'}\left\{\left(\chi\left(\boldsymbol{y}_n^t|\boldsymbol{y}_{n'}^t\right)\right)^\top\left(\mathbf{e}_{nn'}^t\right)\right\}\Big)\,2(1+1/N)/\sigma,$$

where $\chi\left(\boldsymbol{y}_n^t|\boldsymbol{y}_{n'}^t\right)\in\mathbb{R}^M$ is the *Conditional Information Force*, acting on $\boldsymbol{y}_n^{(t)}$ due to $\boldsymbol{y}_{n'}^t$, that is defined as: $\chi\left(\boldsymbol{y}_n^t|\boldsymbol{y}_{n'}^t\right)=\kappa_X\left(\boldsymbol{x}_n\boldsymbol{A}^t,x_{n'}\boldsymbol{A}^t;\sigma\right)\left(\boldsymbol{y}_n^t-\boldsymbol{y}_{n'}^t\right)/N\sigma^2$.

Likewise, we use the stochastic gradient-descent update rule to estimate the Gaussian kernel bandwidth as follows:

$$\sigma^{t+1}=\sigma^t-\mu_\sigma^t\frac{d}{d\sigma^t}\mathrm{var}\left\{v_2\left(\boldsymbol{y}_n^t|\sigma^t\right)\right\}$$

where $\mu_\sigma^t\in\mathbb{R}^+$ is the step size, and σ_x^o is the provided initial guess.
